# Biocatalytic synthesis of lactosucrose using a recombinant thermostable β-fructofuranosidase from *Arthrobacter* sp. 10138

**DOI:** 10.1080/21655979.2020.1739404

**Published:** 2020-03-16

**Authors:** Chunmei Chen, Jieying Deng, Xueqin Lv, Jianghua Li, Guocheng Du, Huazhong Li, Long Liu

**Affiliations:** a Key Laboratory of Carbohydrate Chemistry and Biotechnology, Ministry of Education, Jiangnan University, Wuxi, China; b Key Laboratory of Industrial Biotechnology, Ministry of Education, Jiangnan University, Wuxi, China

**Keywords:** Lactosucrose, thermostable, β-fructofuranosidase, *Arthrobacter* sp. 10138, transfructosylation

## Abstract

As a prebiotics, lactosucrose plays an important role in maintaining human gastrointestinal homeostasis. In this study, a thermostable enzyme from *Arthrobacter* sp. 10138 was screened from six β-fructofuranosidase-producing strains for the lactosucrose production and the coding gene was heterologously expressed in *Escherichia coli* for efficient expression. Recombinant β-fructofuranosidase was purified and biochemically characterized by MALDI-TOFMS spectrometry. The transfructosylation product by this recombinant enzyme was determined to be lactosucrose rather than other oligosaccharides or polysaccharides by HPLC and LC-MS. Efficient extracellular secretion of β-fructofuranosidase was achieved by the optimization of signal peptide and induction conditions. It was found that with the signal peptide torT, the highest extracellular activity reached 111.01 U/mL, which was 38.4-fold higher than that with the OmpA signal peptide. Under the optimal conditions (pH 6.0, temperature 50°C, enzyme amount 40 μg/ml, sucrose 150 g/L and lactose 150 g/L), 109 g/L lactosucrose was produced with a molar conversion ratio of 49.3%. Here the thermostable β-fructofuranosidase from *Arthrobacter* sp. 10138 can be used for efficient synthesis of lactosucrose, and this provides a good startpoint for the industrial production of lactosucrose in the future.

## Graphical Abstract

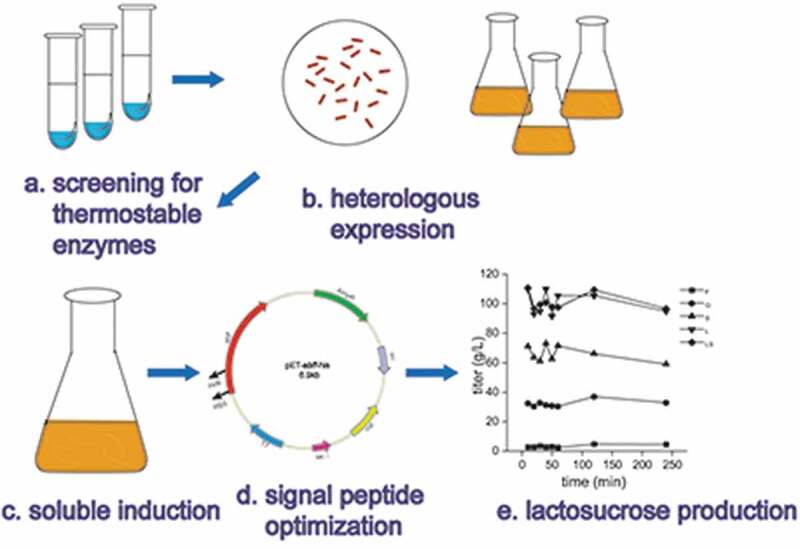

## Introduction

Lactosucrose (O-β-D-galactopyranosyl-(1,4)-O-α-D-glucopyranosyl-(1,2)-β-D-fructofuranoside) is a non-reducing trisaccharide composed of galactose, glucose, and fructose (Figure S1), which can be regarded as a condensate of a molecule of galactose with one molecule of sucrose or a molecule of lactose with one molecule of fructose. Lactosucrose with good solubility and indigestibility is a promising dietary fiber with an ideal prebiotic effect [1]. It was listed in the Foods for Speciﬁc Health Uses (FOSHU) in Japan in 2005 [2] and has wide applications in the food, chemical, pharmaceutical, and feed industries [3–7].

There have been two methods for lactosucrose production [8]. One is to transfer the β-galactosyl group produced by the decomposition of lactose to the C4 hydroxyl group of glucosyl in sucrose by the catalysis of β-galactosidase (EC 3.2.1.23); The other is to transfer the fructose group produced by the decomposition of sucrose to the C1 hydroxyl group at the reducing end of the lactose by the catalysis of β-fructofuranosidase (EC 3.2.1.26) or levansucrase (EC 2.4.1.10). The use of β-galactosidase for lactosucrose production may cause the formation of the other oligosaccharide by-products such as galactooligosaccharide during the transgalactosylation [9], which makes the purification process more difficult. β-fructofuranosidase and levansucrase are classified into the glycoside hydrolase family 68 (GH68) in the CAZy database. The β-fructofuranosidases from *Bacillus* sp. 417 [10] and *Arthrobacter* sp. K-1 [11] have been used for lactosucrose production. The levansucrases from many microorganisms including *Aerobacter levanicum, Bacillus amyloliquefaciens* IFO 15535, *Geobacillus* stea*rothermophilus* ATCC 12980, *Pseudomonas syringae* IFO 14086 [12], *Bacillus natto* [13], *Bacillus methylotrophicus* and *Paenibacillus polymyxa* have been used for lactosucrose production.

Up to now, a large number of enzymes with transglycosylation activity have been identified and characterized. However, in previous studies, the β-fructofuranosidases used for production of lactosucrose were only found in *Bacillus* sp. 417 and *Arthrobacter* sp. K-1. With the increasing commercial interest and industrial application prospects, new β-fructofuranosidases production has drawn extensive attention. On the other hand, the optimal temperature of β-fructofuranosidases used to synthesize lactosucrose was usually below 50°C. If the temperature of the transglycosylation reaction can be increased, the faster reaction rate, higher conversion rate and decreased viscosity of the reaction broth would be advantageously achieved.

In this work, the β-fructofuranosidase screened from *Arthrobacter* sp. 10138 shows good thermostability and good transglycosylation activity. *E. coli* BL21 (DE3) was used as the host strain for the expression of the gene encoding β-fructofuranosidase. The reactions conditions of the purified recombinant β-fructofuranosidase to synthesize lactosucrose were optimized and an efficient process for the lactosucrose production with thermostable β-fructofuranosidase from *Arthrobacter sp*. 10138 was established.

## Materials and methods

### Chemicals, reagents, and bacterial strains

The chemicals standards, including lactosucrose, lactose, sucrose, glucose, and fructose for catalytic reaction and high-performance liquid chromatography (HPLC) analysis were purchased from Yuanye Biology Co., Ltd (Shanghai, China). Yeast extract, peptone, Isopropyl-β-D1-thiogalactopyranosid (IPTG) and other chemicals were purchased from Sinopharm Chemical Reagent Co., Ltd (Shanghai, China). The SanPrep Column Plasmid Mini-Preps Kit and Clon Express^TM^ MultiS One Step Cloning Kit were purchased from Sangon (Shanghai, China). Primer synthesis and DNA sequencing were performed by Shanghai Sangon Biological Engineering Technology and Services Co., Ltd. Bradford assay kit for protein quantitation was purchased from Sangon Biotech Co., Ltd (Shanghai, China). *Arthrobacter* sp. 10138 was purchased from China Center of industrial Culture Collection (http://www.china-cicc.org/). *Aspergillus niger* XQ46 and *Aspergillus oryzae* XQ4617 were generous gifts from Dr. Shen (Jiangnan University, China). The other three strains *Bacillus subtilis* 168, *Aspergillus niger* B (strain information confidentiality) and *Aspergillus oryzae* NRRL 3488 were owned by our laboratory.

### Thermostable analysis

In previous studies, the six strains above mentioned had potentialities to produce extracellular β-fructofuranosidase [14,15]. Therefore, we hoped to screen a thermostable β-fructofuranosidase from these six strains through a catalytic reaction. The screening method was based on the titer of lactosucrose synthesized by β-fructofuranosidase from six different sources at 50°C and 37°C (as a comparison). Growth conditions for the various microorganisms are detailed in Table 1. The collected fermentation broth was further treated to analyzed enzyme activities.

**Table 1.** Strain screened information in this study.

Strain	Classification	Culture temperature(°C)	Medium composition
*Arthrobacter* sp	*Arthrobacter* sp. 10138	30	Glucose 2%, KH_2_PO_4_ 0.2%, Yeast extract 0.15%, (NH_4_)_2_HPO_4_ 0.6%, MgSO_4_ 7H_2_O 0.01%, pH 7.0～7.2
*Bacillus subtilis*	*Bacillus subtilis 168*	37	TB
*Aspergillus niger* A	*Aspergillus nige* XQ4616	27	Yeast extract 1.2%, Peptone 0.8%, MgSO4 7H2O 0.1%, Glucose 4%, (N H_4_)_2_HPO_4_ 0.4%, KH_2_PO_4_ 0.2%, pH = 7
Aspergillus niger B	Unknown	27	Same as above
*Aspergillus oryzae A*	*Aspergillus oryzae* XQ4617	27	Same as above
*Aspergillus oryzae B*	*Aspergillus oryzae* NRRL 3488	27	Same as above

### Recombinant-plasmid construction

The manipulation and isolation of DNA were performed by standard protocols and methods [16]. Since it was the first study to find that *Arthrobacter* sp. 10138 β-fructofuranosidase had high transglycosylation activity, the sequence of this β-fructofuranosidase encoding gene was unknown. We designed primers by blasting the homologous β-fructofuranosidase gene published on NCBI. The primers used for cloning β-fructofuranosidase gene were *bff*-F and *bff*-R (Table 2). The chromosomal DNA of *Arthrobacter* sp. 10138 was prepared as described by Genomic DNA Extraction Kit. The β-fructofuranosidase gene was obtained by PCR amplification with the above chromosomal DNA as a template. Then, the linearized vector pET-22b(+) was amplified by primers pET-22b(+)-F and pET-22b(+)-R. The purified β-fructofuranosidase gene was ligated into the pET-22b(+) expression vector using the Clon Express^TM^ MultiS One Step Cloning Kit, yielding the plasmid pET-22b-*bff*. There was no codon optimization and the gene was designed to contain a C-terminus in-frame 6× histidine-tag sequence by inverse PCR, resulting in the plasmid pET-*bff*-his, which was used as a template to construct the following plasmids.

**Table 2.** Primers used in this study.

Primers	Sequence (5ʹ-3ʹ)
*bff*-F	CCAGCCGGCGAT GGCCGCCACCGA CGCAGCAC
*bff*-R	CAGTGGTGGTGGTGGTGGTGCTTGGCTACTGCCTTGCTGTTCTT
pET-22b(+)-F	GCACCACCACCACCACCACTGAGATCCGGCTGCTAACAAAGCC
pET-22b(+)-R	GGCCATCGCCGGCTG
OmpA-F	ATGAAAAAGACAGCTATCGCGATTGCAGTGGCACTGGCTGGTTTCGCTACCGTAGCGCAGGCCGCTCCGGCCACCGACGCAGCA
OmpA-R	GCTGTCTTTTTCATATGTATATCTCCTTCTTAAAGTTAAACAAAATTATTTCTAG
wsp-F	ACTTTAAGAAGGAGATATACATATGGCCACCGACGCAGCAC
wsp-R	CATATGTATATCTCCTTCTTAAAGTTAAACAAAATTATTTCT
torT-F	ATGCGCGTACTGCTATTTTTACTTCTTTCCCTTTTCATGTTGCCGGCATTTTCGG CTGATGCCACCGACGCAGCA
torT-R	AATAGCAGTACGCGCATATGTATATCTCCTTCTTAAAGTTAAACAAAAT
sufI-F	GGATTGCACTTTGTGCAGGCGCTGTTCCCCTGAAGGCCAGCGCAGCCGGG GCCACCGACGCAGCA
sufI-R	CAAAGTGCAATCCCCGATGCCTGAATGAACTGACGCCGACTGAGTGACATA TGTATATCTCCTTCTTAAAGTTAAACAAAATTATTTC
DsbA-F	ATGAAAAAGATTTGGCTGGCGCTGGCTGGTTTAGTTTTAGCGTTTAGCGCATCGGCGGCGCAGGCCACCGACGCAGCA
DsbA-F	CCAAATCTTTTTCATATGTATATCTCCTTCTTAAAGTTAAACAAAATTATTTC
Dsma-F	ATGGAACGCAGAAGTTTTCTAAAAATGAGTGCAGCCATGGGCTGCGCAGCAACGGTCACTGGCTGTGCCACCGAC GCAGCA
Dsma-R	AACTTCTGCGTTCCATATGTATATCTCCTTCTTAAAGTTAAAC

### Construction of plasmids with different signal peptides

To study the effect of signal peptide on the secretion of β-fructofuranosidase, we fused five different signal peptides to the N-terminus of the β-fructofuranosidase. Take the plasmid pET-*bff-*OmpA construction as an example, overlapping PCR was used to construct the fused plasmids, and the sequences of all primers are listed in Table 2. The OmpA signal peptide was added to β-fructofuranosidase gene through PCR using the primers OmpA-F and OmpA-R. After purifying the amplification products, the recombinant plasmid was transformed into the host strain *E. coli* BL21 (DE3) to induce expression. The other four signal peptides, sufI, Dsma, torT, and DsbA, were also inserted into the expression vector, respectively. The plasmid pET-*bff*-wsp did not contain any signal peptide before the β-fructofuranosidase coding sequence and the strain harboring the plasmid pET-*bff*-wsp was used as the control. The recombinant plasmids mentioned above were sequenced in Sangon Biotech Co., Ltd (China).

### Culture conditions and β-fructofuranosidase purification

Sequence-correct recombinant plasmids were transformed into *E. coli* BL21 (DE3) competent cells. Then single colony was cultivated in Luria-Bertani (LB) medium (10 g/L tryptone, 5 g/L yeast extract and 10 g/L NaCl), followed by Terriﬁc-Broth (TB) medium (12 g/L tryptone, 24 g/L yeast extract, 4 mL/L glycerol, 17 mM KH_2_PO_4_ and 72 mM K_2_HPO_4_) supplemented with 100 μg/mL of ampicillin when necessary in a rotary shaker at 37°C and 220 r/min. When the optical density OD_600_ reached 0.6–0.8, IPTG at a final concentration of 1 mM was added to induce β-fructofuranosidase expression at 28°C for 6 h [17]. To further promote extracellular expression, we induced the overexpression at different IPTG concentrations, times and temperatures.

All purification steps were performed at 4°C. The crude enzyme solution was collected by centrifugation at 10, 000× *g* for 20 min. The crude supernatant was filtered through a 0.22 µm fibrous membrane and then was loaded onto a HiTrap Q HP column (GE Healthcare) for nickel affinity chromatography. The purification process was carried out according to the manufacturer’s protocol (pET His Tag System, Novagen). The targeted protein was eluted by a gradient of 30% B buffer (20 mM sodium phosphate, 500 mM NaCl, 500 mM imidazole, pH 7.0). All purification steps were carried out using an Äkta Purifier System (GE Healthcare, Sweden). The obtained eluate was desalted into 50 mM phosphate (pH 7.0) using dialysis membrane and concentrated using an Amicon Ultra-15 centrifugal filter device with a 10 kDa cutoff membrane (Millipore). All of the samples were purified for the optimization of lactosucrose production.

### β-fructofuranosidase assay, protein determination and SDS-PAGE analysis

To analyze the β-fructofuranosidase activity, 20 mL of culture was centrifuged at 14, 000 × g for 20 min and the supernatant was used as the crude enzyme (the supernatant of fermentation broth) for biocatalysis. The β-fructofuranosidase activity was assayed by using sucrose and lactose as substrates. β-fructofuranosidase activity was determined by lactosucrose titer in a biocatalytic reaction. Namely, 100 g/L substrate and 20 mL of crude enzyme solution (the supernatant of fermentation broth) were mixed in 50 mM sodium phosphate buffer (pH 7.0). The reaction proceeded in a 250-mL shaking flask and incubated on a rotary shaker at 50°C for 12 h. The reaction was stopped by heating it to 100°C for 10 min and followed by centrifugation. The supernatant was recovered for the quantification of lactosucrose by HPLC, as described below. One unit of transfructoslyation activity was defined as the amount of enzyme producing 1 μmol lactosucrose per min by transfructosylation.

Protein concentrations were quantified at 595 nm using a commercial Bradford assay kit (Sangon Biotech Co., Ltd) [18] with bovine serum albumin as a standard. Sodium dodecyl sulfate-polyacrylamide gel electrophoresis (SDS PAGE) in a 10% gel was done by the method of Laemmli [19]. Protein samples were added to the SDS-PAGE loading buffer and heated at 99°C for 10 min. After SDS-PAGE, the gel was stained with 0.05% Coomassie brilliant blue R-250. Finally, we can accurately identify this enzyme by MALDI-TOFMS spectrometry.

### Optimization of pH, temperature, substrate concentration, and enzyme amount

For optimization of all the variables, the reaction was conducted in 1-mL reaction mixture and incubated on a rotary shaker for 20 min. For pH optimization, the reaction was conducted at 50°C in sodium phosphate and citric acid buffer (2.0 − 8.0) containing 150 g/L sucrose and lactose. For temperature optimization, the reaction was carried out at pH 6.0, with temperatures varying between 20 and 80°C. To optimize the substrate concentration, the reaction was conducted at pH 6.0, 50°C, with the substrate concentration ranging from 10% to 30%, w/v. All reactions were carried out with 20 μg/ml purified recombinant β-fructofuranosidase. For enzyme amount optimization, the conditions were 150 g/L substrate, 50°C, and pH 6.0, with the enzyme amount ranging from 20 to 150 μg/ml. The resulting samples were centrifuged, and the supernatant was tested by HPLC.

### HPLC analysis and LC-MS

The concentrations of sucrose, lactose, and lactosucrose were analyzed using HPLC system (Agilent 1260, CA, USA) equipped with an Electrospray detector CAD and Shodex VG-50 4E (4.6 mm id × 250 mm, 5 pm, Shodex, Tokyo, Japan). The column was eluted at a flow rate of 1.0 mL/min at 40°C, and the mobile phase was a mixture of deionized water and acetonitrile (25:75, v/v) [20]. LC-MS analysis was used to identify the lactosucrose structure and the results were analyzed by MASSLynx software.

### Statistical analysis

All experiments were independently carried out at least two replicates, and the results were expressed as mean ± standard deviation (SD). P < 0.05 were considered statistically significant and the results were analyzed by Graphpad Prism 8.

## Results and discussion

### Selection of thermostable β-fructofuranosidase producing microorganisms

**Figure 1.**
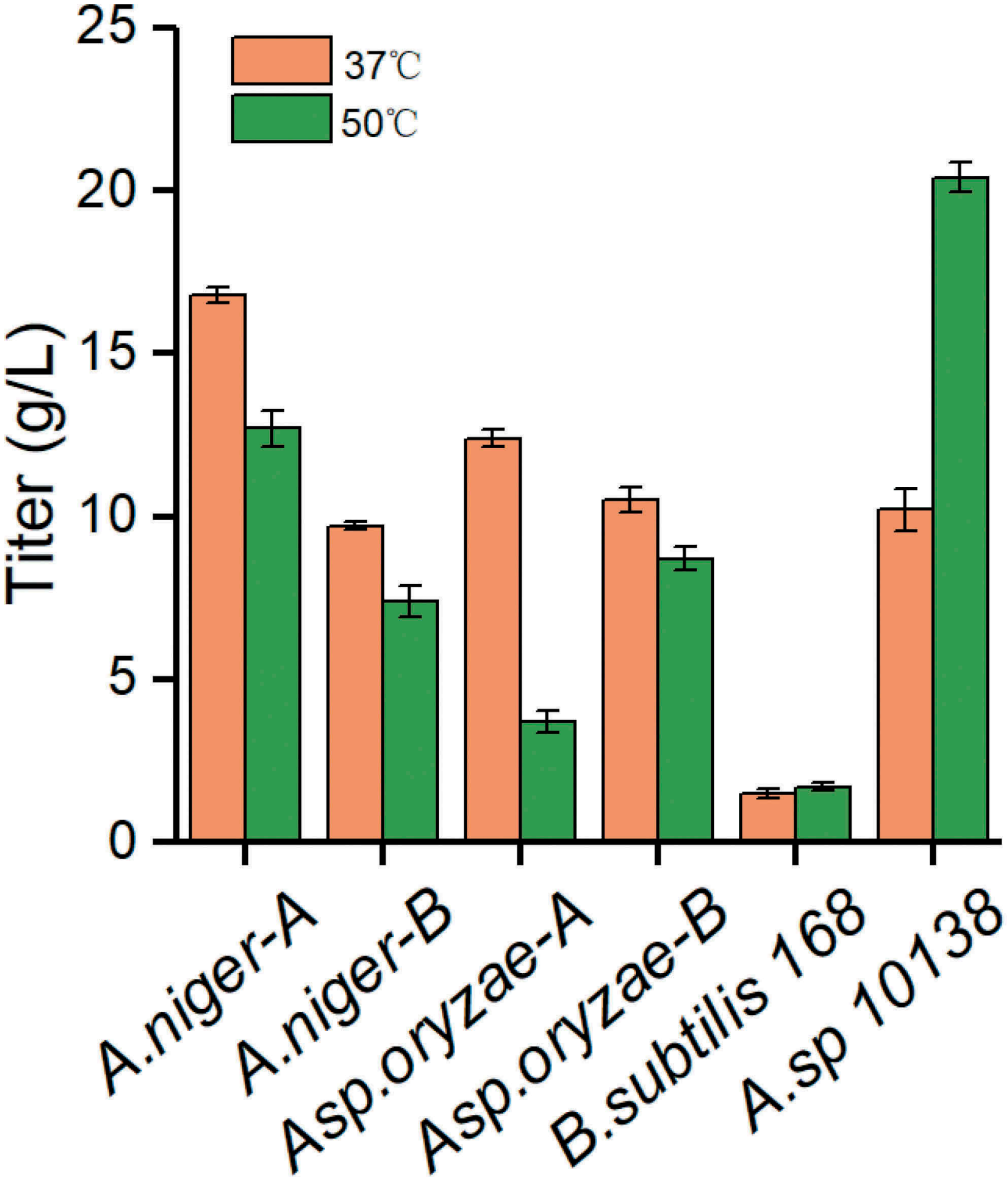
Screening thermostable β-fructofuranosidase from six strains. The strain information is shown in Table 1

Thermostable enzymes, which have the advantages of high reactivity, better yield, high stability, low viscosity and little contamination, are important and highly attractive candidates for industrial. Lactosucrose production by six microorganisms at 37°C or 50°C was investigated using 50 g/L sucrose and 50 g/L lactose as substrates. Figure 1 showed that *Arthrobacter* sp. 10138 β-fructofuranosidase produced the highest titer of lactosucrose as 20.4 g/L at pH 7.0 and 50°C during 12 h. Meanwhile, compared to that at a lower temperature (37°C), the lactosucrose titer did not decrease. Therefore, *Arthrobacter* sp. 10138 was selected as the thermostable β-fructofuranosidase producer for further study.

**Figure 2.**
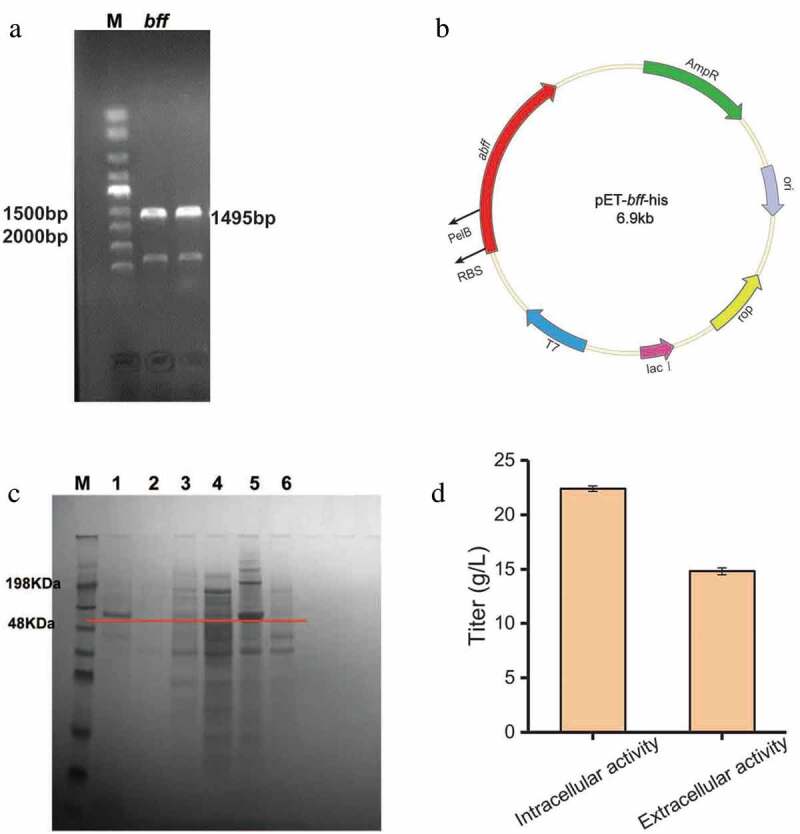
Heterologous expression of β-fructofuranosidase in *Escherichia coli*. (a) PCR analysis of *bff* gene amplified from chromosomal DNA. (b) Construction of expression plasmid pEt-*bff*-his. (c) SDS-PAGE analysis of β-fructofuranosidase. M: protein Marker; 1: recombinant supernatant; 2: blank supernatant; 3: recombinant disrupted supernatant; 4: blank disrupted supernatant; 5: recombinant disrupted lysate; 6: blank disrupted broken lysate. (d) Preliminary enzyme activity assay.

*E. coli* is the preferred host for recombinant protein expression due to its high-level production of heterologous proteins, fast growth and simple culture conditions. In order to improve the enzyme activity and study the production of lactosucrose by β-fructofuranosidase, in the present study the gene encoding β-fructofuranosidase from *Arthrobacter* sp. 10138 was cloned and expressed in *E. coli* BL21 (DE3). The *Arthrobacter* sp. 10138 β-fructofuranosidase gene was successfully cloned using the chromosomal DNA as a template (Figure S2) and the target band was about 1500 bp (Figure 2(a)). The β-fructofuranosidase gene was integrated into the pET-22b (+) vector by a one-step cloning kit and successfully expressed in *E. coli* BL21 (DE3). From the sequencing results of the recombinant plasmid pET*-bff*-his (Figure 2(b)), the total length of *Arthrobacter* sp. 10138 β-fructofuranosidase was 1485 bp, composed of 495 amino acid residues, and the molecular mass was about 56 kDa. By comparing this sequence with the *Arthrobacter* sp-derived β-fructofuranosidase in the NCBI database, we revealed that the homology similarity of β-fructofuranosidase from *Arthrobacter* sp. 10138 with that from *Arthrobacter globiformis* IFO3062 [21] and *Arthrobacter* sp. K-1 [22] was 79.2% and 65.1%, respectively.

### Characteristics of recombinant Arthrobacter sp. 10138 β-fructofuranosidase

The SDS-PAGE results showed that the target protein band (56 kDa) was observed in the supernatant of the fermentation broth, the cell disrupted supernatant and the disrupted lysate, then the inclusion body showed the strongest band (Figure 2(c)). Besides, the preliminary enzyme activity assay of the extracellular supernatant showed that the extracellular enzyme activity was higher than the intracellular enzyme activity (Figure 2(d)). Consequently, the concentration of lactosucrose reached 22.4 g/L when with 50 g/L sucrose and 50 g/L lactose as the substrates.

Furthermore, the MALDI-TOFMS spectrometry analysis showed that the target protein band was levansucrase from *Arthrobacter alpinus* sp. A3 (GenBank: ALV47384.1) with a molecular mass of 56.631 kDa. Due to the absence of *Arthrobacter* sp. 10138 genomic information in the database, MALDI-TOFMS spectrometry detection cannot directly search for the protein from *Arthrobacter* sp. 10138 in the database. However, we compared the partial peptides from enzymatic hydrolysis (Figure S3) with the amino acid sequence of the β-fructofuranosidase derived from *Arthrobacter* sp. 10138, and the sequences were completely consistent. Also, the recombinant enzyme displayed a high identity with levansucrase from *Arthrobacter alpinus* sp. A3 (86.4%). Therefore, we concluded that β-fructofuranosidase from *Arthrobacter* sp. 10138 was successfully expressed in *E. coli* BL21 (DE3).

### Identification of lactosucrose

Lactosucrose detection and quantiﬁcation are generally based on chromatographic methods and we successfully separated the peak of lactosucrose from the peak of substrates and by-products using HPLC system with CAD detector. Based on the chromatogram analysis of five standards including fructose, glucose, sucrose, lactose, and lactosucrose, each was effectively separated with retention time at 7.074, 7.763, 9.608, 11.756 and 14.704 min, respectively (Figure S4).

In order to further determine the presence of lactosucrose, the treated catalytic sample and 1 mg/ml lactosucrose standard were analyzed by LC-MS. The LC-MS results showed that the carbohydrate in the sample had a molecular weight of 504.44 and had the same particle fragments with the standard (Figure S5), and this further verified that the product was lactosucrose. In addition, it was indicated that the enzyme prefers to produce lactosucrose rather than other oligofructoses [23] in the presence of lactose.

### Screening of signal peptides to enhance secretion of β-fructofuranosidase

**Figure 3.**
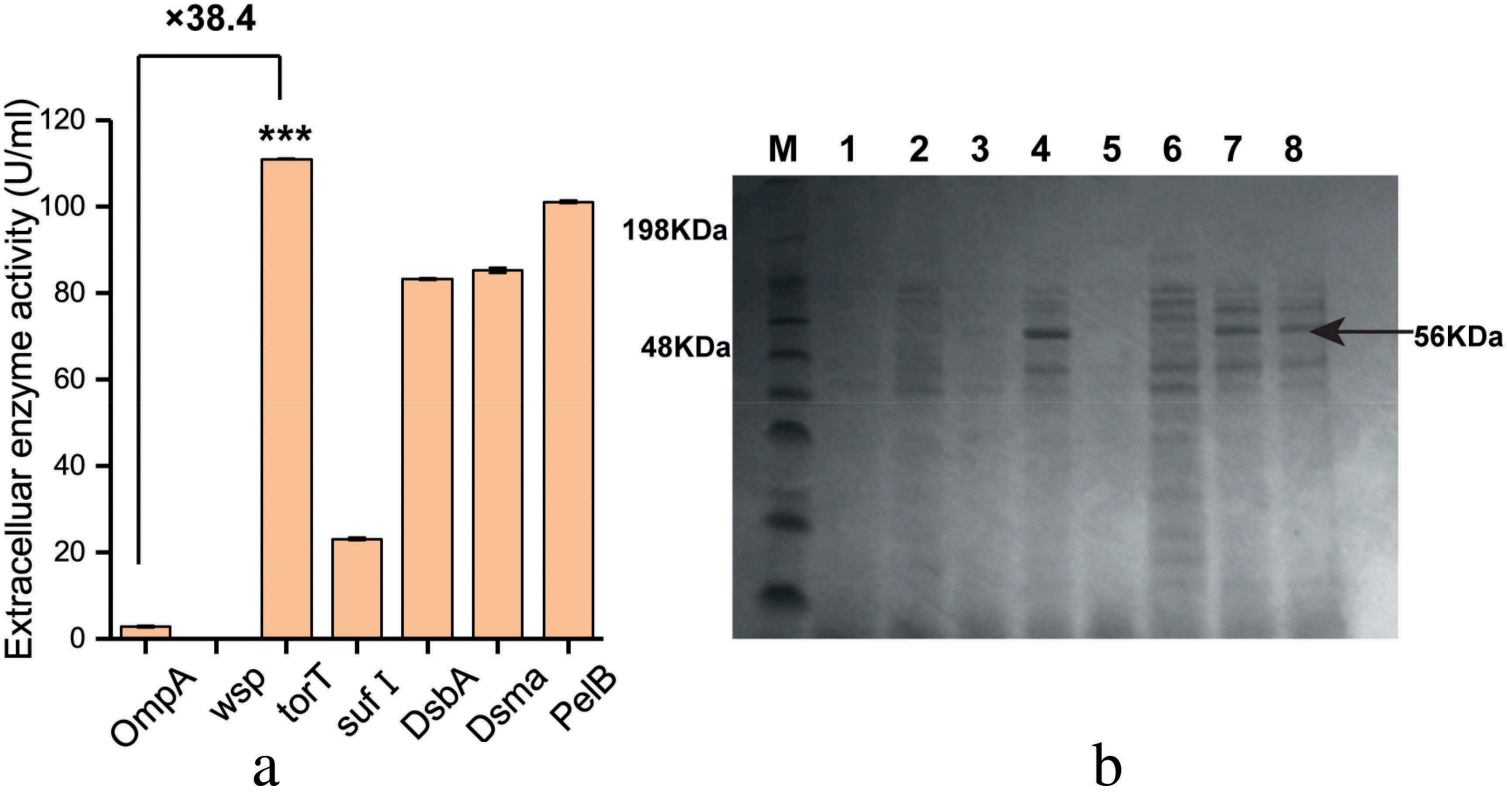
The effect of different signal peptides (SP) on β-fructofuranosidase activity. (a) The extracellular β-fructofuranosidase activity using different signal peptides. (b) SDS-PAGE analysis of extracellular β-fructofuranosidase of the recombinant strains. M: protein marker, 1: blank strain without bff gene, from 2 to 8 represented extracellular protein secretion of OmpA to PelB (a). Differences were determined by 2-tailed Student’s t-test between two groups. Statistical significance is indicated as * for p < 0.05, ** for p < 0.01 and *** for p < 0.001.

To study the effects of different signal peptides on the secretion of β-fructofuranosidase, five signal peptides (Dsma, DabA, OmpA, sufI and torT) were used to replace the original PelB signal peptide, respectively. According to enzyme activity analysis (Figure 3(a)), the extracellular β-fructofuranosidase activity with the signal peptide torT was the highest (111.01 U/mL), and the fusion protein containing OmpA had the lowest extracellular activity (2.89 U/mL). Three strains containing Dsma, DsbA and sufI had higher extracellular β-fructofuranosidase activity than the strain containing OmpA. We also found that the strains lacking signal peptide (wsp) had almost no extracellular enzyme activity.

Due to the specificity of the signal peptide, the secretory pathways of different kinds of proteins in *E. coli* are different. Signal recognition particle (SRP)-dependent secretion pathway, which is characterized by co-translational translocation, helps prevent cytoplasmic aggregation of proteins before secretion [24]. It was found that the torT signal peptide dependent on the SRP pathway had the best secretory capacity. The SDS-PAGE analysis of the extracellular protein secretion was consistent with the results of enzyme activity analysis. In the strains with torT, Dsma and PelB (Figure 3(b)), a band corresponding to the 56 kDa protein was significantly observed in the culture supernatant.

### Optimization of induced β-fructofuranosidase expression in Escherichia coli

**Figure 4.**
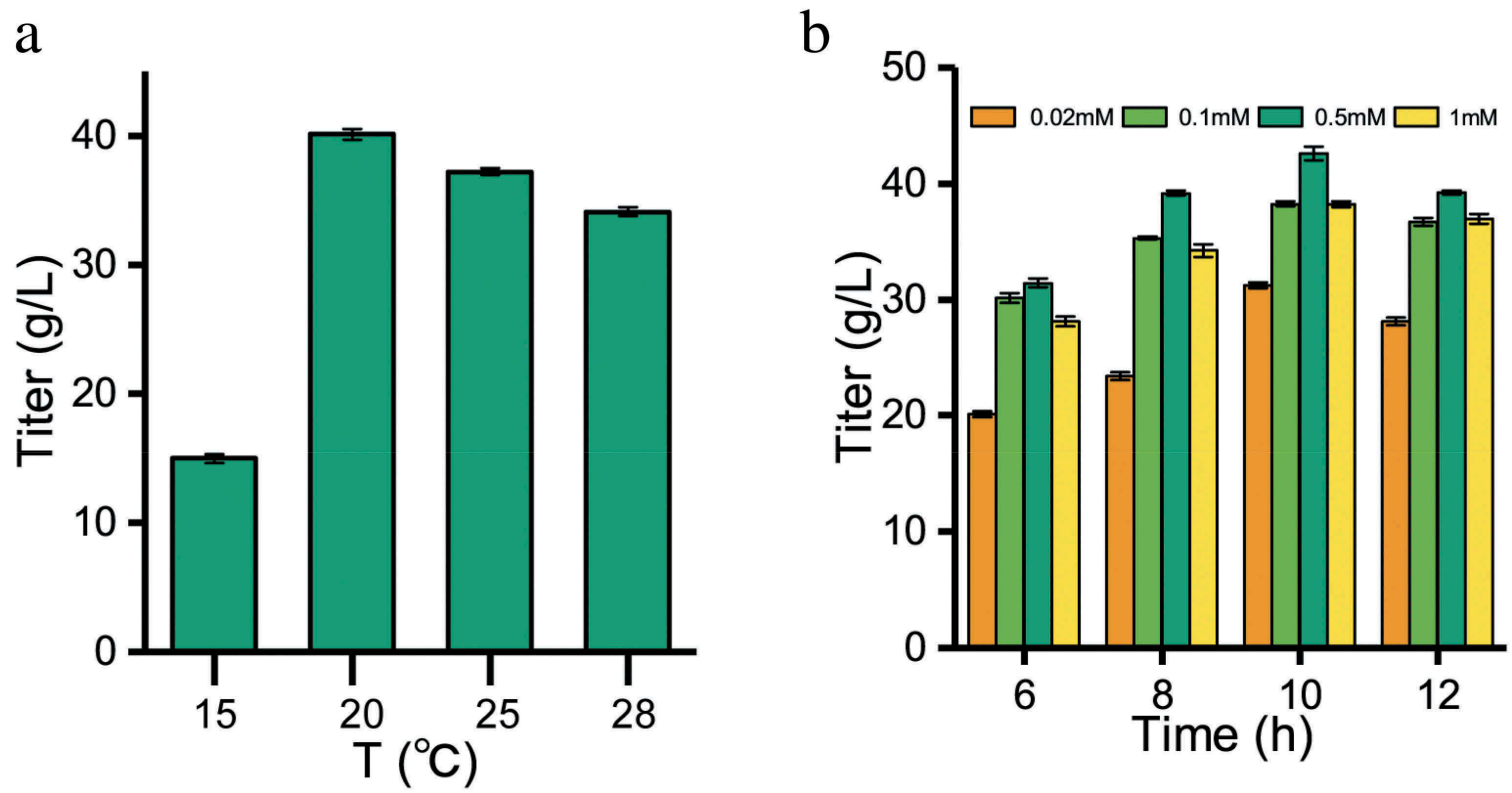
Optimization of induction conditions for soluble expression of β-fructofuranosidase. (a) Effect of temperature on the activities of β-fructofuranosidase. (b) Effect of inducing IPTG concentration and time on the activities of β-fructofuranosidase.

The expression product of the exogenous gene in *E. coli* is usually present in the soluble or insoluble (inclusion body) form [25]. We optimized the induction strategy of β-fructofuranosidase in *E. coli* to assist secretory protein production. As shown in Figure 4(a), the expression of soluble protein was the highest at 25°C, and the enzyme activity was 2.5 times higher than that induced at 15°C. It was indicated that the rate of protein synthesis matched the rate of protein folding to form more soluble proteins at 25°C.

Figure 4(b) showed that the optimal concentration of IPTG was 0.5 mmol/L. When the concentration of IPTG is lower than 0.5 mmol/L, it was not enough to bind the repressor protein, while higher IPTG concentration may have certain toxicity to the cells. After IPTG induction for 6–12 h, both protein and enzyme activity continuously increased over time (Figure 4(b)). With the prolongation of induction time, the accumulation of by-products from the metabolism of *E. coli a*dversely affected the system, and the protein expression and enzyme activity no more increased. At 10 h, the protein expression and enzyme activity were the highest.

### Effect of pH and temperature on the lactosucrose production

**Figure 5.**
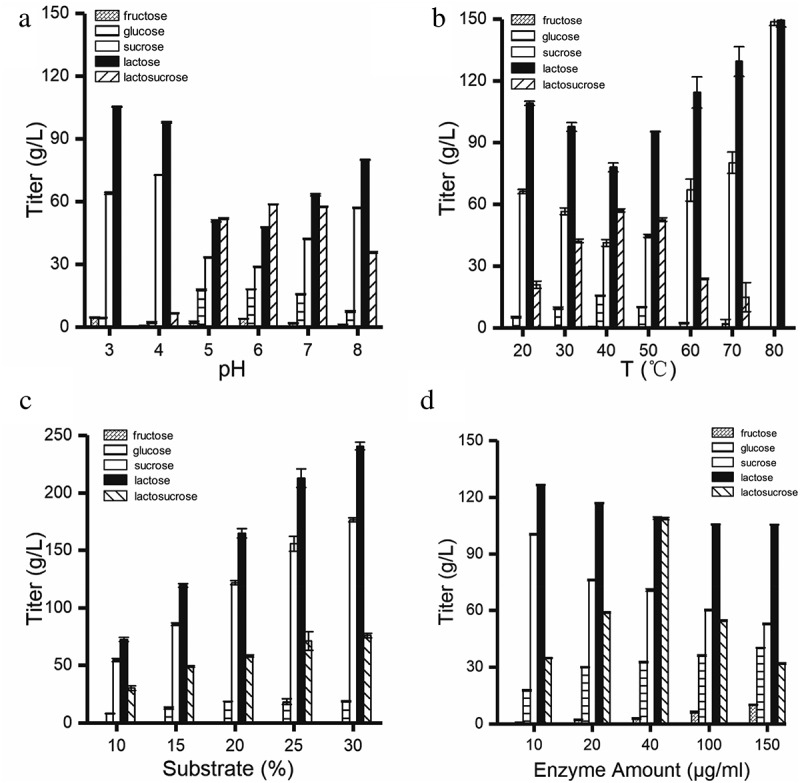
Optimization of four conditions for lactosucrose production. Effect of pH (a), temperature (b), substrate concentration (c) and enzyme amount (d) on lactosucrose production. Values are the means of three replications ± standard deviation.

In order to determine the optimum pH for biocatalysis, the temperature was fixed at 50°C, and the enzyme activity was measured at six gradients of pH between 3.0 and 8.0. As shown in Figure 5(a), the β-fructofuranosidase could efficiently synthesize lactosucrose when pH was between 5.0 and 7.0, and the highest lactosucrose titer of 58 g/L was observed at pH 6.0 (150 g/L each of sucrose and lactose). However, when the pH is lower than 5.0 or higher than 7.0, the titer of lactosucrose decreased significantly, indicating that this recombinant enzyme could not be applied to produce the lactosucrose effectively under these extreme conditions. In general, most of the reported lactosucrose-producing enzymes have an optimum pH between 6.0 and 7.0, except that the *P. aurantica* levansucrase had the highest activity at pH 4.0 [26].

Temperature is an important factor for industrial production of lactosucrose from sucrose and lactose. Increased temperature helps increase the substrate solubility and improve transfructosylation rate [27]. We further evaluated the optimum temperature for the catalytic reaction of β-fructofuranosidase at pH 6.0 and at 7 gradients of temperature between 20°C and 80°C. The maximum titer of lactosucorse reached 60 g/L at 40°C (Figure 5(b)) and the biological lactosucrose production at 50°C was close to that at 40°C. Therefore, the β-fructofuranosidase from *Arthrobacter* sp. 10138 can be a candidate for high temperature biocatalytic production of lactosucrose. In the previous study, the optimum temperature of β-fructofuranosidase from *Arthrobacter* sp. K-1 was 30°C [28].

### Effect of the substrate concentration and enzyme amount on lactosucrose production

Previous studies have shown that the best substrate ratio for levansucrases from *Z. mobiliz, P. polymyxa*, and *B. methylotrophicus* was 1:1 [29], and so we also used equal concentrations of sucrose and lactose. As shown in Figure 5(c), the production of lactosucrose gradually increased by increasing the concentration of each substrate to 300 g/L. In addition, there was a problem that as the concentration of the substrate continuously increased, the concentration of the lactosucrose was continuously increased while the conversion rate was continuously decreased (data not shown). Lactosucrose was produced with the highest conversion ratio at lower substrate concentration (15%, v/w). Therefore, in consideration of the production cost, lower substrate concentration (15%, v/w) may be a better solution.

Influence of enzyme amount on the production of lactosucorse was examined by adding different enzyme amounts of 10, 20, 40, 100 and 150 μg/mL. Figure 5(d) showed that when the amount of enzyme was increased from 10 μg/mL to 40 μg/mL, the titer of lactosucrose significantly increased to a maximum of 108.67 g/L. However, when the amount of the enzyme was higher than 40 μg/mL, the titer of lactosucrose decreased instead, accompanied by the production of two by-products (Figure S4). The stable and slow release of fructose indicated that the hydrolysis reaction continued to occur over time. We suspected that it may be other oligosaccharides produced by the transfructosylation, such as levan. This result was consistent with the investigation of enzyme amount on the transfructosylation product by the levansucrase from *B. goodwinii* [30], *B. methylotrophicus SK 21.002* [31], and *B. subtilis* [32].

The above studies determined the suitable catalytic conditions, that is, pH, temperature, sucrose concentration, lactose concentration and enzyme amount were pH 6.0, 50°C, 150 g/L, 150 g/L and 40 μg/ml, respectively. As the reaction progressed, a maximum titer of lactosucrose reached 109 g/L at 10 minutes (Figure S6), and there was no significant change in titer with the extension of reaction time. We speculated that there may be product or glucose inhibition. Recently, enzyme immobilization [33] and glucose oxidase addition [34] have been used for the continuous lactosucrose production with higher productivity. These techniques may increase the industrialization feasibility of lactosucrose biosynthesis.

Among recent reports, *Brenneria goodwinii* [17] levansucrase and *B.circulans* [35] β-galactosidase produced 100 and 56 g/L lactosucrose, respectively; by comparison, the β-fructofuranosidase from *Arthrobacter* sp. 10138 had a higher turnover yield of lactosucrose. It was interesting that, except for *L.mesenteroides* levansucrase [20], all the purified or crude enzyme produced lactosucrose with titers less than 100 g/L [10,36,37], which were less than those by most of whole cells harboring enzyme activity. Herein, the purified recombinant β-fructofuranosidase from *Arthrobacter* sp. 10138 produced 109 g/L lactosucrose from 150 g/L sucrose and 150 g/L lactose, showing a significantly competitive productivity. Therefore, it was suggested that *Arthrobacter* sp. 10138 β-fructofuranosidase could be used as a novel candidate for lactosucrose production.

## Conclusions

In summary, we screened a thermostable enzyme from *Arthrobacter* sp. 10138, which was more relevant to the industrial requirements. Efficient extracellular secretion was improved through combinatorial strategies including signal peptide screening and optimization of the induction conditions. By means of the thermostable β-fructofuranosidase from *Arthrobacter* sp. 10138, the maximal titer of lactosucrose was 109 g/L after incubation of the purified enzyme (40 μg/mL) with 150 g/L sucrose and lactose for 10 min at 50°C and pH 6.0. The conversion ratio of sucrose and lactose was 49.3% under these conditions. Overall, this work provides a good enzyme candidate for the synthesis of lactosucrose at higher temperature, and the performance of the biocatalyst may be further improved by enzyme engineering in the future.

## Highlights

- A thermostable β-fructofuranosidase was screened from six strains.
- β-fructofuranosidase from *Arthrobacter* sp. 10138 can be a novel candidate.
- The first report about β-fructofuranosidase heterologously expressed in *E. coli*.
- After only ten minutes, the lactosucrose titer reached 109 g/L.
