# Supplementary material for: Biocatalytic synthesis of lactosucrose using a recombinant thermostable β-fructofuranosidase from Arthrobacter sp. 10138
Source: Bioengineered. 2020 Mar 16;11(1):416–27. doi: 10.1080/21655979.2020.1739404 (PMC7161541; doi:10.1080/21655979.2020.1739404)
Supplement: Supplemental Material [file kbie-11-01-1739404-s001.docx]

Table 1. Strain Screened Information in this study.

| Strain | Classification | Culture temperature(°C) | Medium composition |
| --- | --- | --- | --- |
| *Arthrobacter* sp | *Arthrobacter* sp. 10138 | 30 | Glucose 2%,  KH_2_PO_4_ 0.2%，  Yeast extract 0.15%, (NH_4_)_2_HPO_4_ 0.6%，MgSO_4_ 7H_2_O 0.01%，  pH 7.0～7.2 |
| *Bacillus subtilis* | *Bacillus subtilis 168* | 37 | TB |
| *Aspergillus niger* A | *Aspergillus nige* XQ4616 | 27 | Yeast extract 1.2%, Peptone 0.8% , MgSO4 7H2O 0.1%, Glucose 4%,  (N H_4_)_2_HPO_4_ 0.4% , KH_2_PO_4_ 0.2%,  pH= 7 |
| Aspergillus niger B | Unknown | 27 | Same as above |
| *Aspergillus oryzae A* | *Aspergillus oryzae* XQ4617 | 27 | Same as above |
| *Aspergillus oryzae B* | *Aspergillus oryzae* NRRL 3488 | 27 | Same as above |

|  |
| --- |

Table 2. primers used in this study.

| Primers | Sequence (5’-3’) |
| --- | --- |
| *bff*-F | CCAGCCGGCGAT GGCCGCCACCGA CGCAGCAC |
| *bff*-R | CAGTGGTGGTGGTGGTGGTGCTTGGCTACTGCCTTGCTGTTCTT |
| pet-22b(+)-F | GCACCACCACCACCACCACTGAGATCCGGCTGCTAACAAAGCC |
| pet-22b(+)-R | GGCCATCGCCGGCTG |
| OmpA-F | ATGAAAAAGACAGCTATCGCGATTGCAGTGGCACTGGCTGGTTTCGCTACCGTAGCGCAGGCCGCTCCGGCCACCGACGCAGCA |
| OmpA-R | GCTGTCTTTTTCATATGTATATCTCCTTCTTAAAGTTAAACAAAATTATTTCTAG |
| wsp-F | ACTTTAAGAAGGAGATATACATATGGCCACCGACGCAGCAC |
| wsp-R | CATATGTATATCTCCTTCTTAAAGTTAAACAAAATTATTTCT |
| torT-F | ATGCGCGTACTGCTATTTTTACTTCTTTCCCTTTTCATGTTGCCGGCATTTTCGG CTGATGCCACCGACGCAGCA |
| torT-R | AATAGCAGTACGCGCATATGTATATCTCCTTCTTAAAGTTAAACAAAAT |
| sufⅠ-F | GGATTGCACTTTGTGCAGGCGCTGTTCCCCTGAAGGCCAGCGCAGCCGGG GCCACCGACGCAGCA |
| sufⅠ-R | CAAAGTGCAATCCCCGATGCCTGAATGAACTGACGCCGACTGAGTGACATA TGTATATCTCCTTCTTAAAGTTAAACAAAATTATTTC |
| DsbA-F | ATGAAAAAGATTTGGCTGGCGCTGGCTGGTTTAGTTTTAGCGTTTAGCGCATCGGCGGCGCAGGCCACCGACGCAGCA |
| DsbA-F | CCAAATCTTTTTCATATGTATATCTCCTTCTTAAAGTTAAACAAAATTATTTC |
| Dsma-F | ATGGAACGCAGAAGTTTTCTAAAAATGAGTGCAGCCATGGGCTGCGCAGCAACGGTCACTGGCTGTGCCACCGAC GCAGCA |
| Dsma-R | AACTTCTGCGTTCCATATGTATATCTCCTTCTTAAAGTTAAAC |

**Figure legends**

Fig. S1 Structure of lactosucrose

Fig. S2 Nucleotide sequence of the fragment encoding- β-fructofuranosidase from *Arthrobacter* sp. 10138

Fig. S3 Protein identification- MALD and Alignment analysis.

Fig. S4 Product analysis by HPLC with CAD.

Fig. S5 Product analysis by LC-MS. (A) and (B) illustrates LC-MS analysis results of 1 g/L standard sample and catalytic sample.

Fig. S6 Time course of the bioproduction of lactosucrose from sucrose and lactose under the optimized conditions.


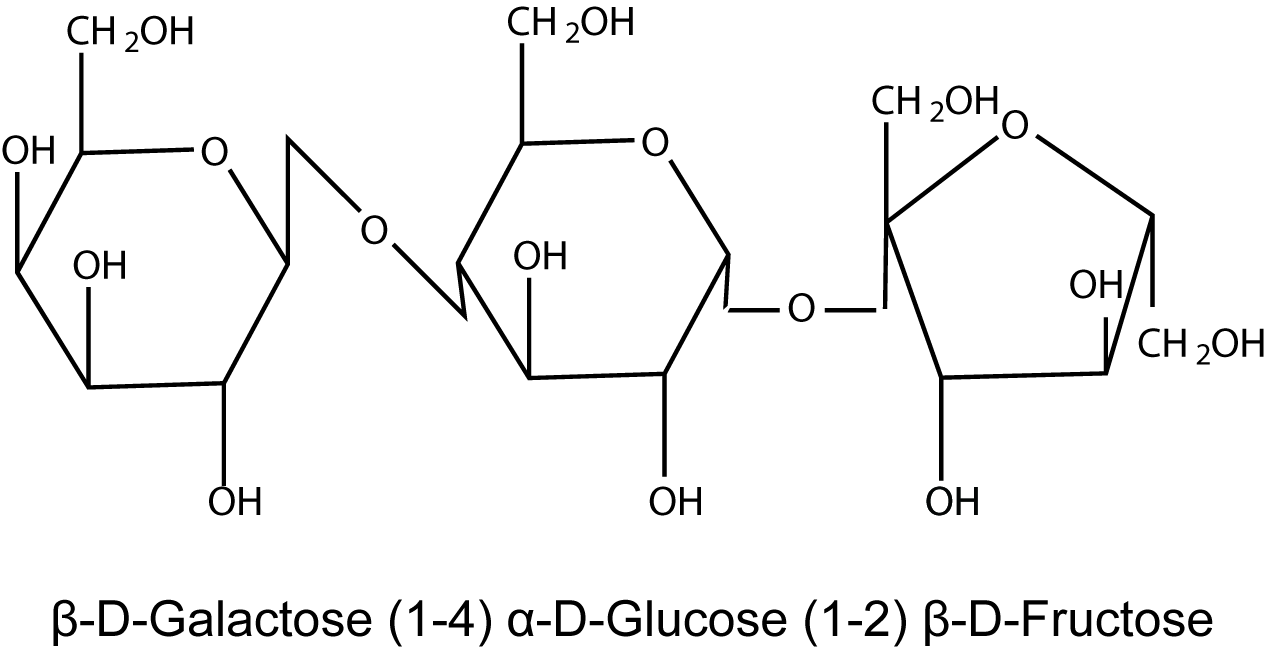


(Figure S1)


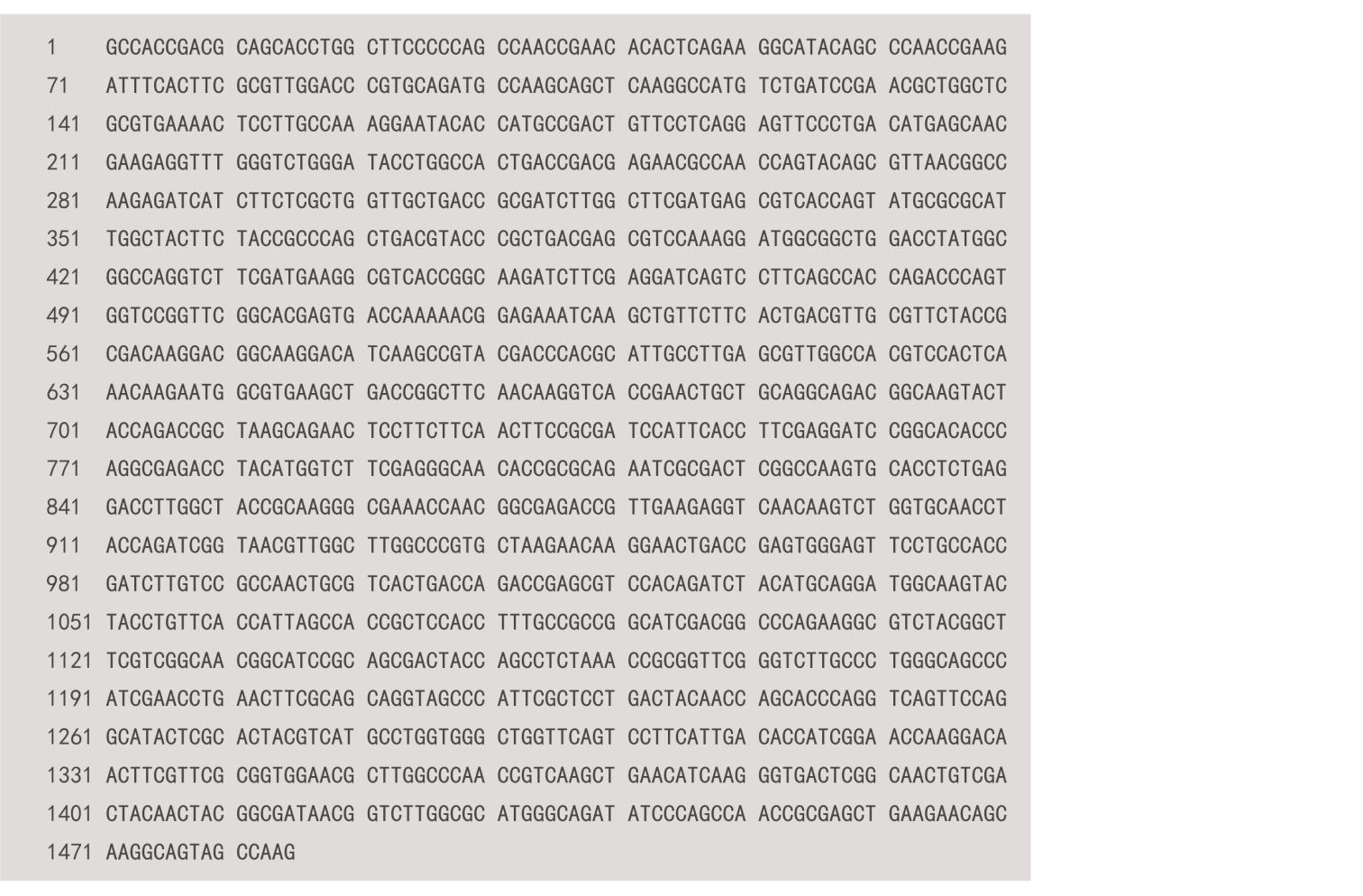


(Figure S2)


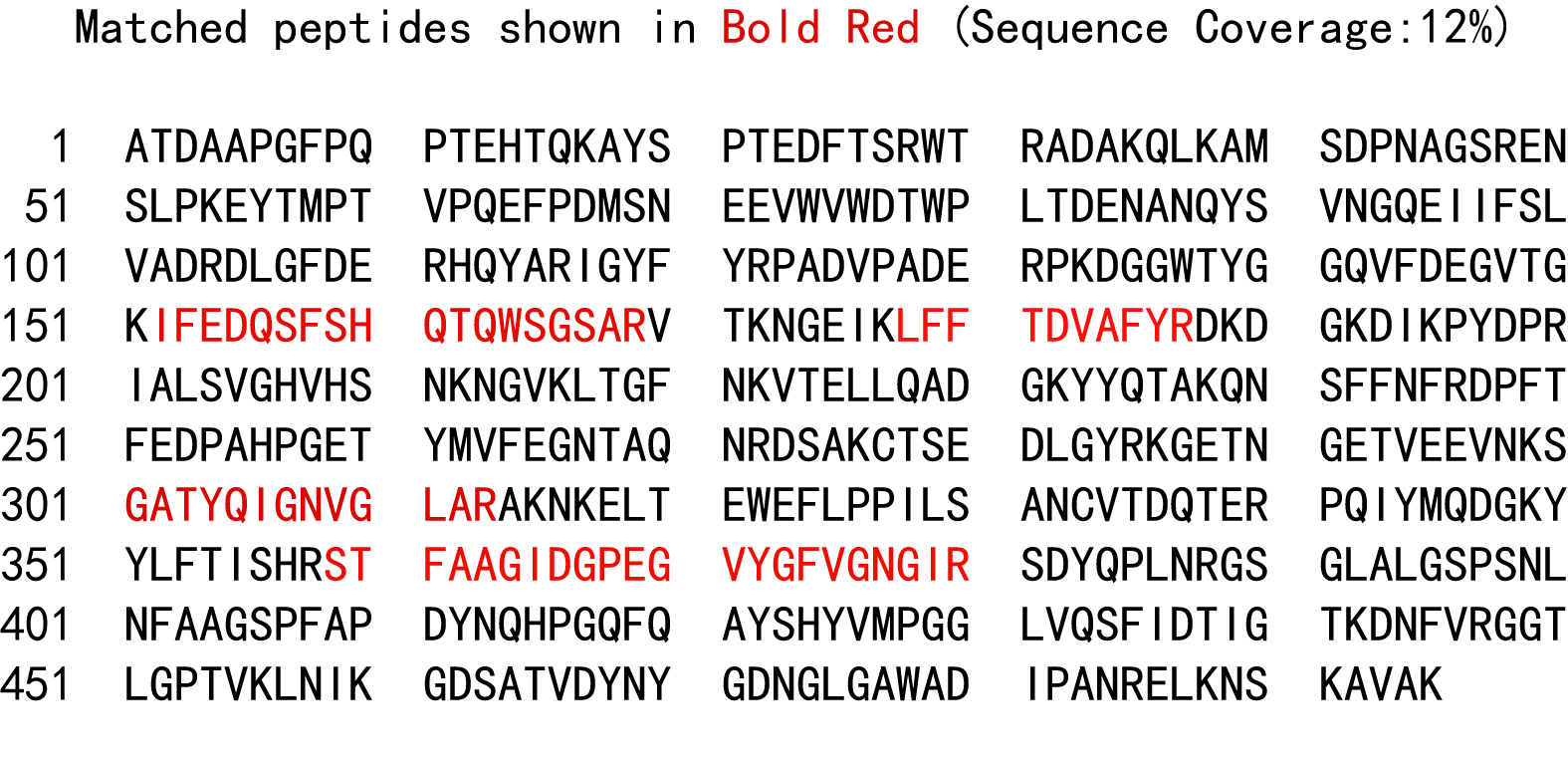


(Figure S3)


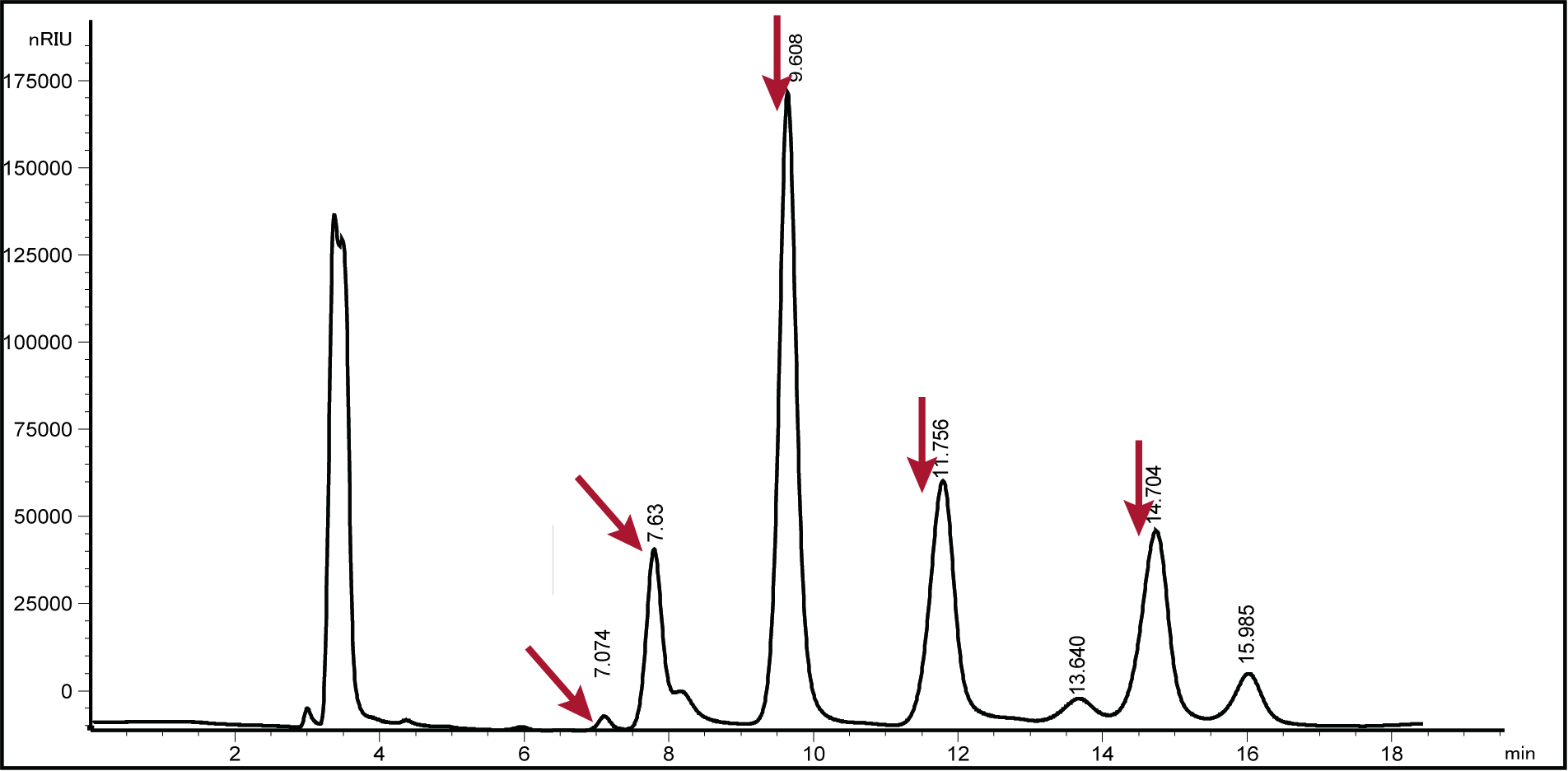


(Figure S4)


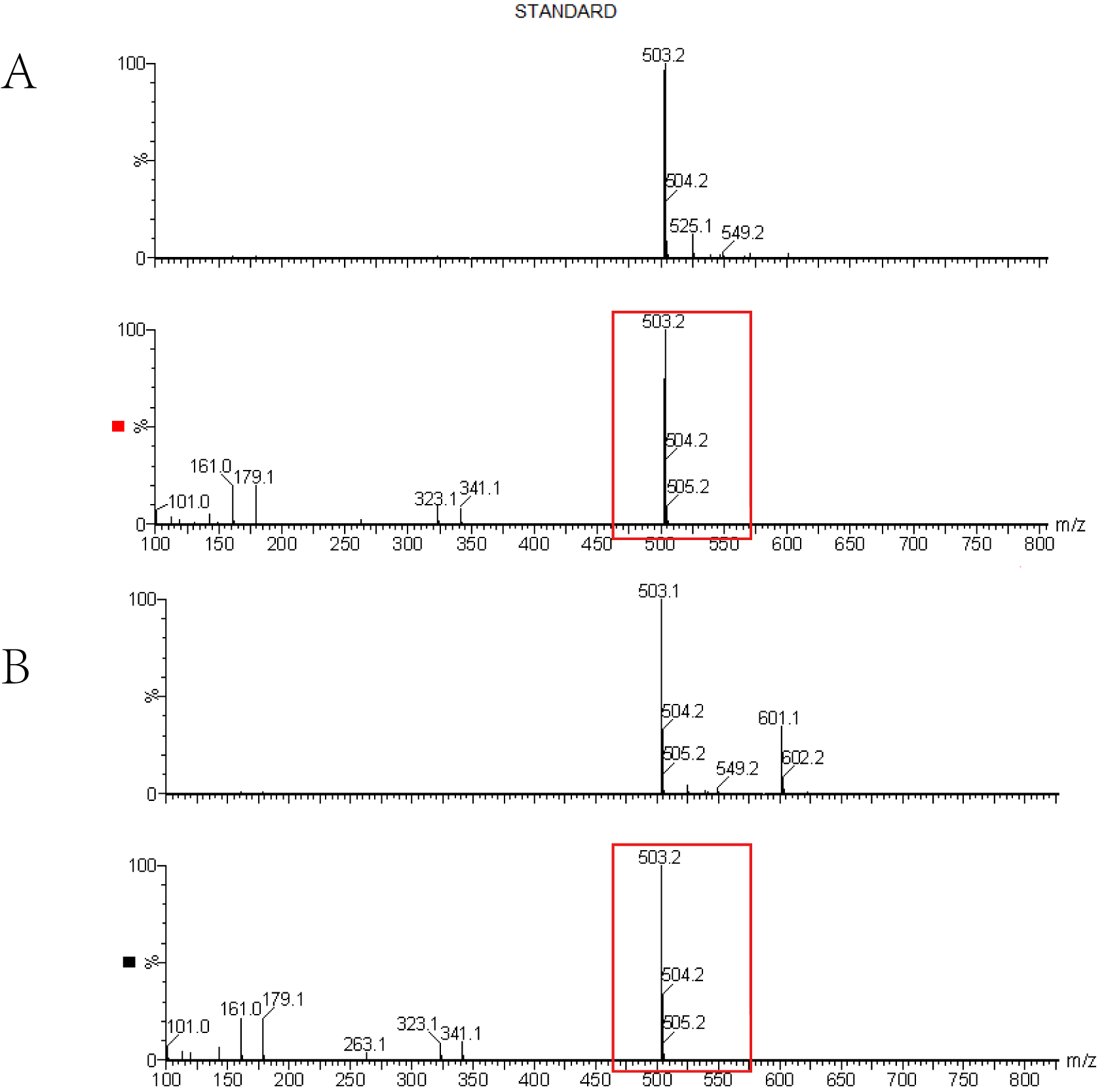


(Figure S5)





(Figure S6)
